# Barriers hindering attendance and adherence to antenatal care visits among women in rural areas in Rwanda: An exploratory qualitative study

**DOI:** 10.1371/journal.pone.0323762

**Published:** 2025-05-21

**Authors:** Olive Tengera, Pamela Meharry, Aimable Nkurunziza, Yolanda Babenko-Mould, Joselyne Rugema, Stephen Rulisa, Laetitia Nyirazinyoye

**Affiliations:** 1 School of Nursing and Midwifery, College of Medicine and Health Sciences, University of Rwanda, Kigali, Rwanda; 2 School of Nursing, University of Illinois Chicago, Chicago, United States of America; 3 Arthur Labatt Family, School of Nursing, Faculty of Health Sciences, Western University, London, Ontario, Canada; 4 Lawrence Bloomberg Faculty of Nursing, University of Toronto, Toronto, Canada; 5 School of Medicine and Pharmacy, College of Medicine and Health Sciences, University of Rwanda, Kigali, Rwanda; 6 School of Public Health, College of Medicine and Health Sciences, University of Rwanda, Kigali, Rwanda; Kandahar University, Faculty of Medicine, AFGHANISTAN

## Abstract

**Background:**

Antenatal care (ANC) improves maternal and neonatal health. However, less than half of pregnant women in sub-Saharan African (SSA) countries, including Rwanda, attend adequate ANC designed to provide routine care and detect and treat early pregnancy complications. This article explores the women’s views on factors that hinder adherence to ANC visits in Rwanda.

**Methods:**

This exploratory qualitative research used in-depth interviews to collect data from 22 pregnant women. Data were recorded, transcribed verbatim, and analyzed using a thematic approach.

**Results:**

A Thematic analysis revealed four themes: a) stigma dynamics, b) sociocultural beliefs and practices, c) lack of partner’s support, and d) Challenges to and at the healthcare setting. Unintended pregnancies, multiparity, and early weaning were identified as reasons why participants delayed attending the ANC. Sociocultural practices and beliefs that place women at the center of domestic chores, cultural misbeliefs, the influence of intergenerational behaviours, and social context were also reported as barriers to attending or adhering to ANC visits. The lack of partner support has been reported as a barrier due to existing family conflicts, domestic violence, competing personal priorities of partners over ANC, and fear of partners of HIV tests. They reported several structural barriers, including attendance and adherence to ANC visits, mandatory requirements before receiving ANC, long distance to the health facility, scattered health services within a health facility, long wait times, and negative attitudes and actions of healthcare providers.

**Conclusions:**

The identified factors hindering pregnant women’s participation in ANC visits are modifiable in progressive countries such as Rwanda. Continued National support could reduce these barriers, increase ANC attendance, and meet the 2030 Sustainable Development Goal 3.

## Introduction

The global maternal mortality ratio in 2020 was 152 deaths/100,000 live births, more than double the 2030 Sustainable Development Goal (SDG) target of 70 deaths/100,000 live births [1]. Maternal deaths are mainly related to pregnancy and childbirth and are primarily preventable during antenatal care (ANC), according to the World Health Organization (WHO) [2]. Globally, while 87% of pregnant women access ANC services with qualified health personnel at least once, only 59% attend four ANC visits. In regions with the highest maternal mortality rates, such as Western and Central Africa and South Asia, only 53% and 49% of pregnant women, respectively [3], attend the minimum number of ANC visits. Until 2016, the WHO recommended four focused ANC visits, and since then, women have been advised to attend at least eight contacts during pregnancy [4].

Antenatal care allows for early detection, diagnosis, and treatment of conditions and diseases affecting pregnancy and may lead to complications during childbirth and postpartum for the mother and newborn [5]. Research has revealed that the ANC package or interventions provided to pregnant women, such as providing supplements, vaccination, and health education, directly and indirectly reduce maternal and perinatal mortality [6]. In Rwanda, the package consists of ANC at health facilities, physical exams, laboratory tests, and health education. Infections such as HIV and malaria that are not detected and treated during pregnancy contributed to almost 25% of maternal deaths and near misses in a local study [7]. The maternal near misses included haemorrhage and sepsis and complications from severe preeclampsia and eclampsia [8]. Although the ANC package content varies depending on the country’s needs, the goal is the same: to reduce perinatal mortality and morbidity.

After considering the various ANC packages, adhering to the number of eight contacts is still challenging, especially in low- and middle-income countries (LMICs)[9,10] Adherence refers to the degree or extent of the woman’s conformity to the WHO recommendations for practice guidelines and the timing, medication dose, and frequency of treatments[5,11]. In this article, ANC adherence refers to the degree to which pregnant women comply with WHO recommendations about the timing, services, and frequency of visits. Studies have shown that sub-Saharan Africa (SSA) and South Asia have the lowest participation in ANC, with most women attending only one visit[3,12,13] In Rwanda, only 47% of women meet the four-visit recommendation[9]. Recent studies have documented the prevalence of pregnant women attending ANC services; however, most used quantitative methodologies and did not sufficiently explore the barriers hindering attendance and adherence to ANC services, thereby limiting a comprehensive understanding of the issue [9]. Therefore, the present study aimed to explore the barriers that hinder pregnant women’s adherence to the recommended ANC visits in the Eastern Province of Rwanda.

## Materials and methods

### Study design

This study used an exploratory qualitative design to determine women’s views on factors that hinder adherence to ANC visits in Rwanda, a developing country in East Africa. This design was most appropriate as it emphasized the subjective nature of the problem. Previous studies had identified the problem quantitatively; we sought to hear directly from pregnant women why adherence to ANC recommendations was challenging. A qualitative descriptive approach is appropriate for studies seeking the opinions of a poorly understood phenomenon[14].

### Study setting

The study was conducted at eight public health centers in two districts in the Eastern Province of Rwanda. The country’s four-level healthcare system ranges from community health posts to national referral hospitals[15]. Public health centers are the next level above health posts and provide ANC services to many pregnant women in the community. Pregnant women with significant complications are referred to the district hospitals. The Eastern Province was selected as it has the lowest proportion of pregnant women attending ANC visits (NISR& International, 2021) and the diversity of the rural health facilities [16].

### Study participants and recruitment

A total of 22 pregnant women were obtained by purposive sampling on days when ANC was provided at the health centres. The primary investigator (OT) checked the appointments in the health center register to identify eligible pregnant women for in-depth interviews. The eligibility criterion was women who attended ANC after 12 weeks of pregnancy, as they were considered late by WHO and national standards. The inclusion criteria were pregnant women 18 or older who attended their first ANC visit after 12 weeks of pregnancy. Potential participants were contacted by healthcare providers working at the health centres and community health workers (CHW) and invited to participate in the study. If the women were agreeable, an appointment for an interview was scheduled at a time when they were available. The study recruitment started on 1^st^ December 2021 and ended on 15^th^ January 2022.

### Data collection

The interview guide was developed rigorously, incorporating insights from a review of relevant literature and expert consultation (S.2). The interview guide included seven semi-structured questions with follow up of probing for deep understanding of the phenomena.

**Interview guide questions**

How do you perceive antenatal care attendance in your community?

What are your perceptions about adherence to antenatal care?

How do you perceive antenatal care services that you receive as a pregnant woman at the health center?

What antenatal care services/packages do you receive at the health center?

What are the barriers that hinder you from antenatal care adherence?

What enables you to adhere to antenatal care?

What are your suggestions or recommendations for improving adherence to antenatal care?

A pre-test was conducted with three participants to increase the guide’s validity, and their feedback informed refinements of question phrasing and flow. During initial interviews, the guide was further revised to address emergent themes, thereby enhancing its effectiveness and ensuring a comprehensive exploration of the participants’ ANC experiences. The primary investigator (PI) was a registered midwife with nearly two decades of experience, and the research assistant (RA) was a registered nurse with seven years of nursing experience. Both had previously conducted qualitative research. Pregnant women who agreed to participate in the study were referred to the PI to arrange the interview.

The PI conducted in-depth, face-to-face interviews with individuals in a private room in the health center. Data saturation was assessed iteratively during the data collection process. Saturation was deemed to have been reached when successive interviews revealed no new codes or themes, and the same patterns began to recur. By the 20th interview, thematic redundancy was evident, and no novel insights were observed in the final two interviews. To maintain confidentiality, only the PI and RA were present during data collection, and the audio recordings were identified with only a study number. The participants were informed about the study’s purpose, participation was voluntary and anonymous, and they could withdraw at any time. Participants were also aware that the interview would be audio recorded, and the RA would be present to document observations. Interviews were conducted in the Kinyarwanda language and lasted 35–50 minutes.

### Description of the analysis process

The recorded interviews were transcribed verbatim in Kinyarwanda. Subsequently, the 22 transcripts were compared with the audio recordings to ensure accurate transcription. After verification, the transcripts were translated into English by the PI, who is fluent in both Kinyarwanda and English. Inductive analysis was used to identify, describe, and interpret themes from the raw data. The thematic analysis was done using Atlas TI software (version 7) (S.1) by the PI and manually using Braun and Clarke’s qualitative analysis[17] by the second investigator (PM). After independently reading and becoming familiar with the data, the investigators used the following separate approaches to data analysis.

The thematic analysis using Braun and Clarke’s approach[17] included systematically generating initial codes for all data, recognizing potential themes, mapping, refining, and naming the themes, and identifying potential quotes as examples to support the findings. The research team noted the content codes and discussed their views to ensure credibility. These were initial codes that were discussed to achieve similar understandings and interpretations. The code was then deduced in terms of subthemes, which were then manually classified as emerging themes by drawing similar topics and thoughts together. The subthemes and themes were revisited several times until a meaningful interpretation was achieved.

#### Trustworthiness.

The researchers followed the trustworthiness principles of credibility, confirmability, dependability, and transferability to ensure the validity and rigor of the present investigation [18]. Only participants who met the research inclusion requirements were selected to be in the study to maintain credibility. Member checking was achieved through calling some participants to verify findings their feedback was incorporated into the final analysis to strengthen the trustworthiness of the results. Consistency was achieved between the two investigators, who analysed the data independently by cross-checking codes and themes during the process. A third member of the research team assisted in resolving discrepancies to reach a consensus, further supporting the inter-related reliability. Reflexivity was used throughout the process, beginning with the research team’s expertise and experiences in midwifery and nursing. To increase credibility and integrity, the research team had regular discussions and iterative reflections to verify that interpretations remained grounded in the data.

### Ethical consideration

The study protocol was approved by the Institutional Review Board of the University of Rwanda, College of Medicine and Health Sciences (CMHS/IRB/223/2021). Permission to conduct the study was obtained from the ethics committee of the two district hospitals. The participants were assigned a unique study code to safeguard anonymity and confidentiality. No personal identifying information was collected. Participants were assured of privacy and anonymity related to the collection of sensitive information prior to signing the consent form. The transcripts were given a study number at the time of enrolment. Digital data were encrypted and stored on password-protected devices, while hard copies were secured in locked storage.

## Findings

Twenty-two pregnant women voluntarily consented to be interviewed for the study while attending ANC in Eastern Province community health centers. No one refused to participate in an interview. We present the participants’ characteristics and four themes and subthemes.

### Characteristics of the participants

Most of the women were married (95.5%), and their ages ranged from 21 to 45 years. Eighteen women (81.8%) had finished primary school, and three (13.6%) had secondary education, while only one (4.5%) had not attended school. Most of the women (77.3%) were multiparous with two or more children. Eighteen women (81.8%) were farmers, and many (68.2%) were at the lower economic level.

### Themes

Four themes emerged: a) stigma dynamics, b) socio-economic and cultural beliefs c) lack of partner support, and d) Challenges to and at the healthcare setting.

#### Stigma dynamics.

Pregnant women reported that stigma is one of the barriers to attending ANC visits. This stigma is associated with an unplanned or unwanted pregnancy or early weaning of the young infant from breastfeeding due to a short interval between births.

**The stigma attached to unintended pregnancies.** Adolescent girls and women with unintended pregnancies are often stigmatized in society, causing delays or preventing the attendance of ANC. In this study, the participants faced shame and feared being laughed at while attending ANC visits. Some participants reported a delay in ANC visits due to the shame and embarrassment of others seeing them during pregnancy.

For example, one participant reported what happened to a colleague: “*By attending ANC, everyone will know that she is pregnant, and she will be ashamed. An unmarried young girl will give birth at her family’s house and then fear being stigmatized and dismissed by the family”.* (HC2-P4) In a similar context, another participant explained that adolescents first hide pregnancies from their parents and other family members to avoid stigma in their families. “... *you hide the pregnancy from your parents… and then they will know…at about the time of delivery”.* (HC3-P7)

**Stigma attached to multiparity and early weaning.** Many women who had previously given birth faced barriers to accessing ANC; they may attend only one time and then give birth. Women who gave birth when their youngest child was still an infant were among those who avoided ANC for fear of stigma and humiliation from within the community due to a very short interval between pregnancies and early weaning. Furthermore, women having many births may become depressed, and this condition may lead them to be late or not attend ANC, and then there is the added stress of where she gives birth. For example, a participant mentioned:

*“My neighbour became pregnant when her youngest child was only nine months old. She delayed her first antenatal care visit to hide her pregnancy and ultimately gave birth during her second visit, fearing that people would judge her for getting pregnant again so soon”.*(HC2- P6) Another participant recalled that women from large families might avoid attending antenatal care services to escape negative comments and judgment from members of their community. For example, one said*, “This pregnant woman is often giving birth as if she has a funded project!”* (HC1-P1)

Women who weaned their children early [before two years] hide their pregnancies to avoid negative comments from colleagues. In this context, a participant stated: “*When people see that you got pregnant while you have a very young child, they start talking about your ability to care for all of these children”.*(HC2- P6)

#### Socio-economic and cultural beliefs.

Some participants reported that delaying ANC attendance was caused by socio-economic and cultural beliefs that place women at the center of domestic chores, cultural misbeliefs, intergenerational behaviours, and social context.

**Gender roles.** Participants described that Rwandan women are the heart of the family. They must juggle multiple responsibilities that could hinder their individual ability to attend an ANC visit. In addition to the domestic chores, many women have to dig daily for food on the farm to support the family. They fetch water for drinking and washing, raise goats, and feed cattle, among their many activities. Not only they are the homemakers and raising and educating children, but women may also have an outside job, such as a farmer. For various reasons, it can be difficult for those with many children or women who work away from home to attend the ANC. For instance, one participant noted:

“*When you have school children, you are in charge of their books, clothes, and all other necessities for them to pursue their studies. Therefore, sometimes women miss out on reaching the health center or nearest hospital for an ANC visit”.* (HC4-P3)

Many rural residents lack the adequate time and energy to think about their unborn babies and decide not to travel to health facilities due to their multiple responsibilities and the overwhelming number of activities.

“*Men treat women as if they are not human beings. They use us for heavy work, exhaustive work. However, when you are pregnant, you can say that you are tired and have no energy. However, after delivery, they do not care about you again”.* (HC2- P1)

**Economic situation**: A woman’s ability to initiate ANC was highly dependent on community-based health insurance (CBHI), which covers the cost of pregnancy care. If women do not have CBHI, they often delay ANC until the third trimester or fail to attend due to financial constraints. One participant noted, “... *she comes late due to the struggle to find money to pay for health insurance”*.(HC4-P2) Another participant recalled, “*In my community… there was a woman in the lowest economic level… she has eight children and finds it … very hard to afford CBHI*”.(HC2- P6) Women had financial problems that hindered ANC attendance, not only CBHI payments but also the money to buy an ANC card that is needed to write information, including the next appointment, as the following participant reported: *“I had a challenge of making my ANC appointment due to lack of money to buy an ANC card. I waited for it”*.(HC1- P6)

**Cultural misbelief:** Some women do not attend ANC on time or at all due to cultural misbeliefs, traditions, or ignorance. Some participants recounted how others hid their pregnancies to protect themselves from being targeted by witchcraft, which was a significant barrier to attending in the first trimester. Some mentioned the presence of harmful beliefs and superstitions in their community, including fears that such practices could hinder the growth of the fetus or cause complications during labor and delivery. “*Women then perceive a need to be careful so that no one knows that they opt for antenatal care”.*(HC2- P4) Similarly, another participant added,

“*I wanted to come early but thought I might meet someone I know. We don’t want people from our village to see us coming to ANC early in pregnancy, as they can harm our pregnancy… and this is why we are late… we are hiding from those people”.* (HC4-P4).

Some women were noted to follow some ancient practices to overcome these misconceptions, which delay their ANC attendance until after the first trimester or go to a traditional healer for informal support. For example, a participant said: “... *I know some women who meet liars advocating for traditional healers and their medicine. They go there instead of the health centers where they would get the correct information, assessments, and treatment”.*(HC4-P3).

**Influence of intergenerational** **behaviours.** Some compare this generation to their mothers who delivered at home; if there was no problem with the pregnancy, they could wait until the baby was at term. One participant recalled: “*My mother-in-law told me she came [to the health center] at eight months and no one blamed her. Therefore, there was no reason to rush my husband, and that the husband [her son] will arrange an antenatal visit when he has time “.(*HC3-P7). Similarly, another participant confirmed this: *“… A husband might say, ‘There is still time; don’t rush me. In the past, pregnant women gave birth at home without attending antenatal care at health facilities, and they still had healthy children.* (HC3- P5) Women who attended early ANC were perceived in the community to need more visits due to pregnancy complications.

**Social context.** Given the social nature of pregnancy, women like to look smart for their ANC visits and may wait until their husbands have bought them new clothes. A participant explained: “A *woman may feel like she has nothing to wear, which would make her ask: ‘How am I going to an ANC visit while other mothers have new clothes?’ Sometimes she stays at home”.* (HC1 P3)

In addition, some participants reported having difficulty attending the initial visit with their husbands. The women explained that sometimes it is challenging when their husbands work far away and only return home on weekends. If the husband is absent, the wife has to go to the local leader to request permission to attend ANC without him. Consequently, some women may delay attending, or others may prefer traditional healers. One participant reported,

“*If you cannot get to the health center with your husband for the right information, assessment, and the appropriate treatment…, you may meet a pregnant woman talking about a traditional healer who helped her,and you may opt to go to there instead….”.(*HC4-P3)

#### Lack of partner support.

Participants reported that ANC visits were delayed due to a lack of support from their partners due to family conflict, domestic violence, conflicting priorities, and fear of HIV testing. Some women prefer to be alone without communicating with others, depending on their situation.

**Family conflicts.** Women reported that misunderstandings and conflicts with their husbands can delay ANC visits. If the couple has conflict, women may hide their pregnancy from their husband and, therefore, would not be accompanied to their first ANC visit. They may also hide any evidence of attending care. A participant mentioned, “*These women... may hide the ANC card that they received at the health facility or even hide their pregnancy from their husbands”.*(HC2- P2)

Sometimes, these conflicts lead to domestic violence, which results in delays or avoidance of ANC. Some participants were afraid of their husband’s reactions to an unwanted pregnancy, particularly those on a family planning method to prevent pregnancy. They explained that they were unsure how they got pregnant while using a contraceptive method, although they may not have used it properly, such as missing an oral contraceptive one day.

Furthermore, a husband may ask his wife, where did that pregnancy come from? when the husband had previously sent his wife to the family planning clinic. If the method fails, the husband may become angry at his wife and state, I am not the father of this baby. The wife may then decide not to attend ANC and instead deliver at home*.* In some extreme cases, the husband may accuse the wife of adultery, and the pregnant woman will decide not to attend ANC services. One participant asked, *“How can I seek ANC when my husband denies the pregnancy? Who will be the father of the child on the birth registration? I may decide not to attend the health center”*(HC1 P3).

**Partners’ competing priorities.** Participants reported that some partners may not prioritize ANC and avoid responsibilities for the family expenses. The partner might be the only one with an income and may buy personal items instead of paying for his wife’s ANC expenses. For example, “*A wife may talk about going for an ANC visit, and the husband says, ‘he doesn’t have the money and what he has is reserved for beer’”*. It is difficult for some women to raise a family and pay ANC-related costs such as the CBHI, transportation, and admission fees. Consequently, they prioritize their children’s needs over their own. One participant described the following situation:

“*You suspect that your colleague is seven to eight months pregnant. When you ask why she came late to the ANC... she replies, ‘I am jobless. I don’t have CBHI, even for my other children. How can I use all that money for an unborn child while his siblings are hungry?’”.* (HC2- P5)

**Fear of HIV testing among partners.** A husband often refused to accompany his wife to the initial ANC visit as they feared the HIV test and a positive result. “*They told us to bring our husbands on the first visit because some women test positive for HIV/AIDS. When he is not there, there is a conflict. Some husbands refused to come, asking, ‘How can I handle my wife if I test positive?’”.*(HC1-P1)

#### Challenges to and at the healthcare setting.

Participants reported several challenges to attending and adhering to the ANC appointment setting, including the long distance to the facility, scattered health services at the site, long wait times, and negative attitudes and actions of healthcare providers.

**Distance to the health facility:** Many women reported that living far away and lacking access to transportation, such as a motorcycle or bicycle, deterred women from attending ANC visits. Some women arrived late and tired after traveling long distances on foot and climbing mountains to reach the health facility. Knowing that they had to do the same journey home after a day at the health facility made matters worse. This exhausting situation led many women to attend ANC only once during their pregnancy. For example, a participant noted, “... *if I were pregnant and lived far away in a small village near the river (lake), plus the weakness of carrying this pregnancy… I would not be able to attend all ANC visits”.*(HC2-P4) In this similar context, another participant confirmed this by saying: “*There is an issue of long trips and bad roads that make it not easy to even use motorcycles for transport”.(*HC4-P2).

**Scattered services at the health center.** Pregnant women reported frustration in accessing the services they needed at the facility, such as the laboratory, restrooms, and other essential services. Feeling weak and hungry while trying to locate the various buildings made this even more challenging. For example, one participant recounted, “*Let me tell you another challenge that delays people in the health center... the long walk to the bathrooms”.*(HC2-P2). In a similar vein, another participant added:

*“When a woman is pregnant, she may not have anything to eat, and she may be weak. They make you go to various services in different buildings until you become exhausted and frustrated. Additionally, there are no toilets nearby”.*(HC1-P3)

**The attitudes and actions of healthcare providers.** The attendance of pregnant women at ANC visits was negatively affected by nurses’ and midwives’ attitudes and actions. Participants criticized some of the HCP’s behaviours, such as shouting, scolding, pettiness, and talking to them as if they were children. For instance, one participant explained what happened to her colleague:

“*The husband had not shown up, and so the nurse did not communicate with the pregnant woman; she told her nothing. The woman said, ‘Please forgive me and provide antenatal care to me’. Instead, the nurse chased her away. The pregnant women left dissatisfied and discouraged* [without receiving ANC]*. The pregnant woman replied, ‘If I am back after my pregnancy has grown, you will shout at me that I am late’”.* (HC1-P3)

In some cases, the long waiting time in health centres can be attributed to misconduct or disorganization by HCPs. Some HCPs were observed making lengthy phone calls during work hours, causing delays in care and likely discouraging pregnant women from returning for follow-up visits as the following woman recalled:

“*Since these phones came out (smartphones), the person responsible for providing care may take calls and delay care for some hours… while we wait for her help. You feel embarrassed, but you must calm down and wait for her! That makes us not want to return”.* (HC1-P4)

A woman sent home without HCP support may turn to traditional healers. Pregnant women can become discouraged when faced with disrespectful care and share that experience with others, as displayed in the following excerpt.

“*The healthcare provider may talk badly to a pregnant woman by saying, ‘Come back tomorrow because you paid without asking me.’ The pregnant woman may never return and will tell her story to a friend… or her neighbour!”*(HC3-P5)

## Discussion

This qualitative study explored the barriers that prevent pregnant women from attending and adhering to ANC visits at public health centres in Rwanda. The findings of this study highlight the significant sociocultural barriers that hinder timely antenatal care (ANC) attendance among women, particularly the stigma attached to unintended pregnancies, multiple pregnancies, and early weaning. Previous research has shown that stigma associated with unintended pregnancies can discourage women from seeking timely maternal healthcare due to fear of judgment from family and community members[19,20]. Stigma was a key theme related to barriers to accessing maternal health care for pregnant adolescents in a qualitative study conducted in South Africa where adolescent pregnancy perpetuated a culture of non-disclosure and shame, inhibiting a high-risk population from seeking much-needed ANC[21]. In many societies, women who become pregnant outside of marriage or without prior planning often experience social exclusion, which may lead to delays in seeking ANC services[22,23]. Such stigma can result in poor maternal and neonatal outcomes due to late detection of pregnancy complications. Furthermore, multiparous woman and those who were weaned off early while breastfeeding their previous children also did not adhere to ANC visits. This is because they did not disclose their pregnancy to avoid being mocked. Similarly, a study conducted in Sudan showed that grand multiparas may face stigma from HCPs or society due to their high parity. This can discourage them from seeking ANC [24]. It is essential to provide supportive and non-judgmental care for all pregnant women especially those who are vulnerable and to stigma.

Intergenerational influences play a crucial role in shaping women’s health-seeking behaviours. In many communities, older women, including mothers-in-law and grandmothers, exert substantial influence over pregnancy-related decisions, often discouraging ANC visits in favour of traditional practices[25]. This is particularly relevant in patriarchal societies where younger women have limited autonomy over their healthcare choices[26]. Social norms that prioritize domestic responsibilities over personal healthcare further contribute to ANC delays, as women are expected to fulfil household duties before seeking medical attention[27]. Additionally, traditional beliefs surrounding pregnancy, such as the notion that discussing a pregnancy too early invites misfortune, further discourage early ANC attendance[28]. Other women reported a delay in ANC visits due to sociocultural practices and beliefs. These practices place women at the centre of domestic chores, cultural misbeliefs, intergenerational behaviours, and the social context.

In the present study, we found that women who became pregnant while using the FP method delayed attending ANC visits, as they were on contraception and did not think they were pregnant. In addition to feeling ashamed to attend visit, they were frustrated by the failure of the FP methods. This finding is similar to that of a study conducted in Ethiopia, where women with unintended pregnancies were less likely to seek ANC services [24]. Additionally, the failure to use FP methods can cause conflict with the husband, resulting in a negative impact on the access and use of ANC and increase chance of a home birth. The husband may deny the pregnancy and not accompany his wife to the first ANC visit. The findings highlight the importance of counselling and education about the efficacy of FP methods. This approach ensures that women who become pregnant while using contraception are not ashamed to attend ANC visits. Spousal involvement in pregnancy interventions should target families planning pregnancies to ensure that men are fully involved and ready to support pregnant mothers throughout their pregnancy journey.

Participants reported delays in ANC visits due to socio-economic and cultural beliefs and practices. Social norms prioritizing domestic responsibilities over personal healthcare contribute to ANC delays, as women are expected to fulfil household duties before seeking medical attention[27]. Women have multiple tasks and responsibilities at home, including caring for their children and husbands and often lack the time and energy to think about their unborn babies and travel to a health facility, which is similar to studies conducted in Australia and Uganda [29,30].

Another challenge to attending ANC is the CBHI healthcare package, which adversely affects the timing and attendance at ANC visits. Some women could not afford the CBHI earlier in the pregnancy, instead they bought food for their children. A meta-analysis of over a million people in 20 LMICs found that CBHI plans significantly improved outpatient healthcare use [31].

Our findings revealed that women hide their pregnancy and delay attending their first ANC visit due to fear of witchcraft similar to the findings of other studies in Malawi, Gambia, and Zambia [32–34].This deterrent prevents pregnant women from initiating ANC early and attending recommended visits. Our study also found that some women rely on family members for advice rather than health care providers. This finding is similar to a systematic review in rural African countries where women may seek traditional medicine or spiritual practices instead of accessing evidence-based healthcare services [35].

Interestingly, in this study, due to the social nature of pregnancy, some women waited for their husbands to buy them stylish dresses to ensure that they look suitable when meeting others on the way to and from the health facility. There is a need for community awareness among women about the importance of attending prenatal care in healthcare facilities rather than seeking informal support. These social contexts should be considered when suggesting interventions to increase ANC attendance and adherence among women in rural areas and reduce potential harm.

We discovered that women sometimes hide their pregnancy even from their husbands due to conflicts. This delays initiation into the healthcare system as the husband is required to accompany his wife for HIV and syphilis testing. Conflict can lead to intimate partner violence (IPV) and affect ANC attendance and adherence, which is supported by other studies conducted in Guinea and Spain[36,37]. Healthcare providers should strive to identify and provide appropriate support to women who suffer from IPV, as it is harmful to both the mother and fetus. IPV is associated with delayed or no ANC, substance abuse, depression, and low infant birth weight[38]. Providing IPV prevention interventions to couples planning a pregnancy can help reduce the risk of IPV before it occurs during pregnancy when women are most at risk.

In this study, women reported that competing personal priorities over ANC is a significant barrier. For example, women reported that their husbands prioritize solving their issues over helping them attend ANC and feeding the family. This dilemma led to women not adhering to recommended ANC visits, even not attending at all, due to lack of financial support from their husbands. These findings corroborate those of a study conducted in Pakistan, which revealed that wives and husbands have numerous expectations of each other shaped by cultural norms and gender roles[39]. In addition, women reported that due to the lack of support from their husbands to support their family, for example, by putting food on the table, they end up using their own money to feed their children instead of securing Community Based Health Insurance (CBHI) or paying transport fees, which adversely affects the timing and attendance of ANC. Similar findings were reported in a meta-synthesis of qualitative studies showing that the lack of resources increased the risk of non-attendance and physical danger during travel[13]. Other studies have shown that the costs of attending ANC include transportation costs. Unemployment can be particularly difficult for women who live far from health facilities or who have limited financial resources[40,41]. Women’s empowerment is needed to help them generate income to pay some costs while attending ANC visits.

The study participants reported several structural barriers to attendance and adherence to ANC visits, including mandatory requirements before receiving ANC, long distance to the health facility, scattered health services within one health facility, long wait times, and negative attitudes and actions of healthcare providers.

Although it is mandatory for women to come with their husbands at the first ANC visits to get tested for HIV[42], some women reported it as a challenge, especially when their husbands work far from home and are only available during the weekend, while ANC services are closed or for adolescent mothers who are not legally married and are sometimes abandoned by their partners. These women added that they are sent back to the community to bring a copy of confirmation from their local leaders that their husbands could not attend on weekdays. As a result, some may not come back, as they can seek the support of traditional healers. Second, in this study, it was reported that some husbands fear being tested for HIV, which prevents them from accompanying their wives. Other studies reported similar findings [43,44].

The study participants reported several barriers that delayed or prevented attendance and adherence to ANC visits, beginning with the journey to the health facility. The distance to the facility could be a significant deterrent for pregnant women, particularly if it is physically enduring, dangerous, or there are limited financial resources for transportation [41]. Distance may not be the issue only for the woman, but getting the husband to the first ANC visit could be a problem, with services only available on weekdays when he is likely working.

The access of pregnant women to ANC services was affected by passing through many different buildings to seek services such as laboratory tests and health insurance verification, causing delays in care. Similarly, studies conducted in Australia and Uganda showed that women experiencing disadvantage faced a wide range of barriers to accessing and receiving ANC, including spatial fragmentation of services [30,45]. This finding suggested that addressing spatial fragmentation, such as the relocation of essential services in one building, is essential to improve the attendance of pregnant women in the ANC. The long wait times at the health facility also discouraged pregnant women from attending ANC appointments. This may be related to inadequate human resources, where staff often struggle to provide timely and quality care. These findings support the findings of studies conducted in northern Jordan, South Africa, and Nigeria [46–48].

Although effective communication between HPs and pregnant women is crucial to promoting attendance, this study revealed that provider behaviours, such as rudeness and negligence, discouraged attendance at ANC services. This finding is similar to that of a study in Malawi [32] and a systematic review conducted in East Africa, which revealed that disrespectful and abusive maternity care is a sign of poor treatment that influences women’s choice to attend maternal services[26]. A study conducted in Ethiopia suggested that written policies and the provision of training to healthcare providers have the potential to improve respectful maternal care[49]. Clear and comprehensive information about the importance of ANC, the benefits of specific interventions, and the potential risks can positively influence women’s attitudes and encourage attendance[36]. This finding shows that the application of respectful maternity care by HPs while caring for pregnant women, even throughout the continuum of care, may improve patient-centred ANC and attract women to adhere to ANC-recommended visits[50]. Waving some mandatory requirements can help some women increase participation in the ANC but also women’s empowerment is needed to help them generate income to pay some costs while attending ANC visits.

### Strengths and limitations of the study

The main asset of this study included data from expectant mothers who had trouble getting to appointments with the ANC. Qualitative methods recorded respondents’ perceptions and experiences in their regular social contexts. Additionally, because this study was carried out in only two districts in the eastern province, the results cannot accurately represent what other Rwandan communities have experienced. Based on this study, interviewing men would be an excellent idea for future research. This study offers a comprehensive understanding of the numerous barriers that prevent women from attending ANC visits; however, additional research focusing on various geographic locations would benefit evidence-based policy making.

## Conclusions

The study showed that the main barriers to attending and adhering to recommended ANC visits were multifaceted, from individual to system level. These barriers include stigma associated with unintended pregnancy, multiparty and early weaning, sociocultural practices and beliefs, and a lack of partner support. Interventions to increase awareness of the importance of attending ANC should target individuals, couples, and communities to address these barriers. Structural barriers, including mandatory requirements before receiving ANC, long distance to the health facility, scattered health services within one health facility, long wait times, and the negative attitudes and actions of healthcare providers, were also reported to hinder attendance and adherence to ANC visits. Policies must be reviewed to eliminate the structural barriers women face when attending ANC. Providing training to nurses and midwives to remove their negative attitudes toward women would be a step toward increasing attendance and adherence to ANC.
